# A novel *PML::RARA* fusion in acute promyelocytic leukemia: a case report and literature review

**DOI:** 10.3389/fonc.2026.1784103

**Published:** 2026-04-14

**Authors:** Jia-Ping Chen, Jun-Jie Hou, Xiao-Yong Chen, Yi-Xuan Cao, Peng Ke, Ji-Hao Zhou, Li-Na Hu

**Affiliations:** 1 Department of Hematology, The Second Clinical Medical College of Jinan University, Shenzhen People’s Hospital, Shenzhen, Guangdong, China; 2 Department of Hematology, Shenzhen People’s Hospital (The Second Clinical Medical College of Jinan University, The First Affiliated Hospital of Southern University of Science and Technology), Shenzhen, Guangdong, China

**Keywords:** APL, FLT3-ITD, PML::RARA fusion transcripts, RARA rearrangements, real-time quantitative PCR assays, RNA sequencing, WT1 mutations

## Abstract

Acute promyelocytic leukemia (APL) is a distinct subtype of acute myeloid leukemia defined by the t(15;17)(q24;q21)-derived *PML::RARA* fusion. However, a small subset of patients harbor cryptic or atypical *RARA* rearrangements that escape detection by routine real-time quantitative RT-PCR (qRT-PCR). We report a 34-year-old man presenting typical APL in whom repeated testing for the canonical long, short, and variant *PML::RARA* transcripts yielded negative results. RNA sequencing subsequently identified a previously unreported in-frame fusion linking *PML* exon 8 to a 58–base pair–deleted *RARA* exon 3. The resulting chimeric transcript retained the *PML* coiled-coil domain as well as the DNA- and ligand-binding domains of *RARA*, suggesting preserved sensitivity to retinoid-based therapy. Consistent with this prediction, induction therapy with all-trans retinoic acid (ATRA) and arsenic trioxide (ATO) resulted in achievement of complete molecular remission. Molecular relapse occurred three months after premature discontinuation of maintenance therapy, underscoring the leukemogenic potential of this novel fusion. This observation expands the molecular spectrum of APL and highlights the potential value of incorporating RNA sequencing into the diagnostic workflow for morphologically suspected but PCR-negative APL.

## Introduction

Acute promyelocytic leukemia (APL) is a biologically and clinically distinct subtype of acute myeloid leukemia characterized by a unique molecular pathogenesis, clinical presentation, and therapeutic vulnerability. It typically affects middle-aged adults and presents with life-threatening bleeding complications, most notably disseminated intravascular coagulation (1). APL is driven by the t(15;17)(q24;q21) translocation, which generates the *PML::RARA* fusion protein and results in differentiation arrest of myeloid precursors. This molecular lesion represents both the central pathogenic driver and the therapeutic target of all-trans retinoic acid (ATRA) and arsenic trioxide (ATO) (2).

To date, multiple variant APL-associated rearrangements involving *RARA* have been described, including *PLZF, NPM1, NUMA1, STAT5B, PRKAR1A, FIP1L1, BCOR, NABP1, TBL1XR1*, GTF2I, *IRF2BP2*, and *FNDC3B*, collectively accounting for approximately 1–2% of APL cases (3). Nevertheless, current diagnostic algorithms rely heavily on the detection of canonical *PML::RARA* fusion transcripts by standardized real-time quantitative PCR assays (4, 5). Rare or atypical breakpoints may therefore escape routine molecular screening, potentially leading to diagnostic delay and increased early mortality. Comprehensive characterization of uncommon *RARA* rearrangements is essential to refine diagnostic workflows and ensure timely initiation of targeted therapy.

## Case description

**Figure 1:**
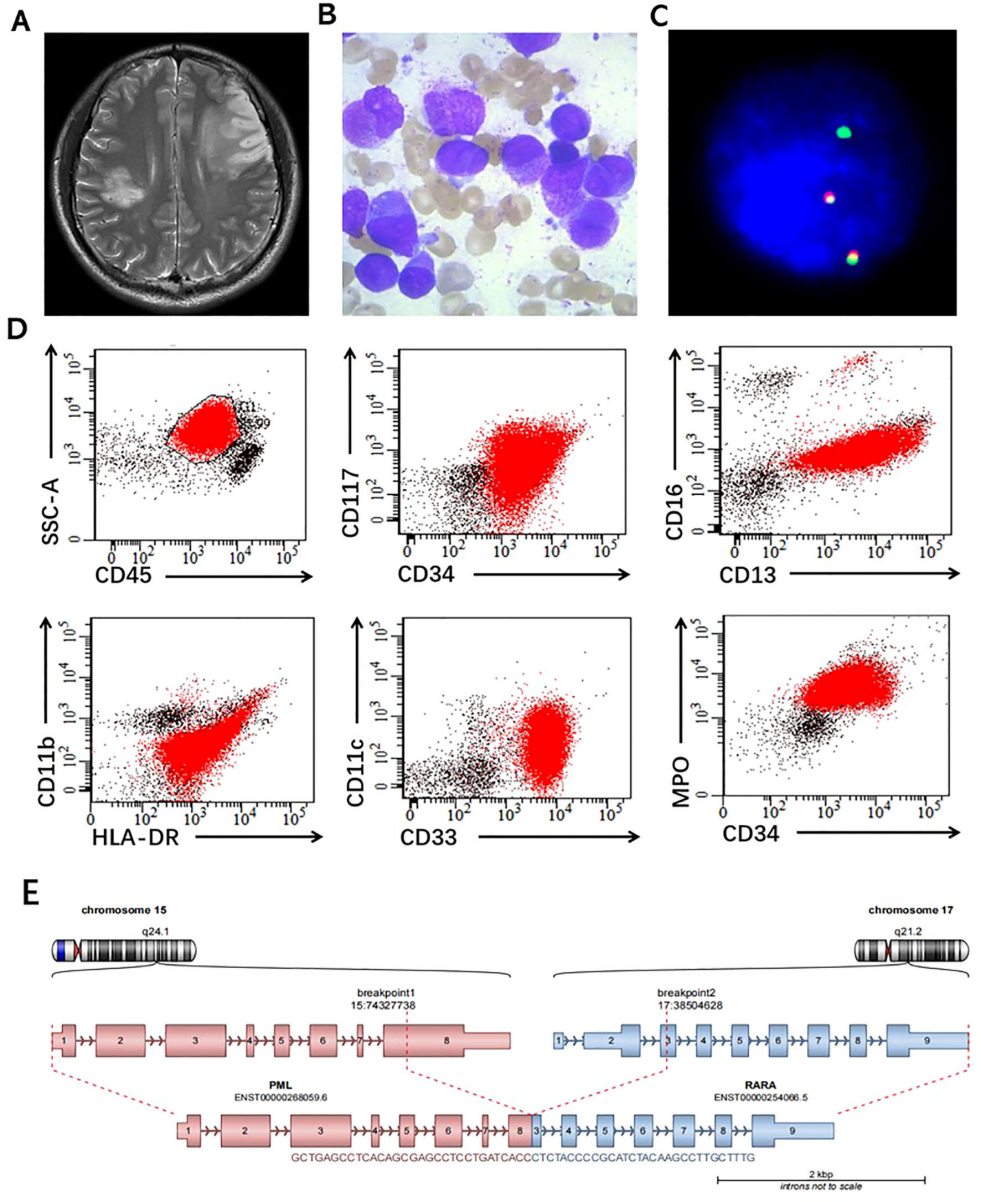
Integrated characterization of the acute promyelocytic leukemia case. **(A)** Brain MRI; **(B)** Bone marrow cytology; **(C)** FISH analysis for PML-RARA; **(D)** Flow cytometry immunophenotyping (CD45⁺ blasts expressing CD34, CD117, CD13, CD33, MPO, with partial CD11b, CD11c, CD16 and HLA-DR); **(E)** Molecular schematic of the t(15;17) translocation generating PML-RARA fusion.

A 34-year-old man was admitted in February 2024 after the acute onset of aphasia, with incidental detection of marked leukocytosis. Initial laboratory evaluation revealed a white blood cell count of 47 × 10^9^/L, hemoglobin of 70 g/L, and a platelet count of 26 × 10^9^/L. Coagulation studies demonstrated hypofibrinogenemia (1.18 g/L), prolonged prothrombin time (18.4 s), and markedly elevated D-dimer levels (>20 µg/mL FEU), consistent with overt disseminated intravascular coagulation. Brain magnetic resonance imaging revealed multiple hyperintense lesions involving the bilateral frontal and parietal lobes as well as the left cerebellar hemisphere, consistent with central nervous system involvement (**Figure 1A**).

Bone marrow aspirate demonstrated 98% hypergranular promyelocytes with abundant faggot-shaped Auer rods, consistent with FAB M3 morphology (**Figure 1B**). Fluorescence *in situ* hybridization (FISH) demonstrated rearrangement of the *RARA* locus (**Figure 1C**). Flow cytometric analysis showed that 93.7% of nucleated cells expressed CD33, CD13, CD117, CD64, CD38, and cytoplasmic MPO, with partial expression of CD56 and CD9, and absence of CD34 and HLA-DR, supporting a diagnosis of APL (**Figure 1D**). Despite the characteristic morphologic and immunophenotypic features of APL and evidence of *RARA* rearrangement by FISH, repeated molecular testing failed to identify any known *PML::RARA* fusion transcripts.

Multiplex real-time quantitative RT-PCR assays targeting all canonical *PML::RARA* isoforms repeatedly yielded negative results. To resolve this diagnostic discrepancy, RNA sequencing was performed and identified a previously unreported in-frame fusion linking *PML* exon 8 to a 58–base pair–deleted *RARA* exon 3, a pattern consistent with prior reports of atypical breakpoints escaping PCR detection (**Figure 1E**). Concomitant mutations involving *FLT3-ITD* and *WT1* were also detected, placing the patient in a very high-risk molecular category. Patient-specific primers targeting the unique fusion breakpoint were subsequently designed to enable sensitive molecular monitoring of minimal residual disease.

Induction therapy was initiated promptly with ATRA and ATO, in combination with daunorubicin and cytarabine-based chemotherapy (DA), given the patient’s high leukocyte count and central nervous system involvement, in accordance with European LeukemiaNet recommendations (6). By day 35 of induction, quantitative molecular analysis demonstrated an approximately 3-log reduction in fusion transcript burden. Complete morphologic remission with molecular negativity was achieved after induction thearpy. The patient subsequently received consolidation therapy and intrathecal prophylaxis. Maintenance therapy with ATRA and compound realgar–indigo tablets was initiated, and bone marrow evaluation confirmed sustained complete molecular remission (CMR).

**Figure 2:**
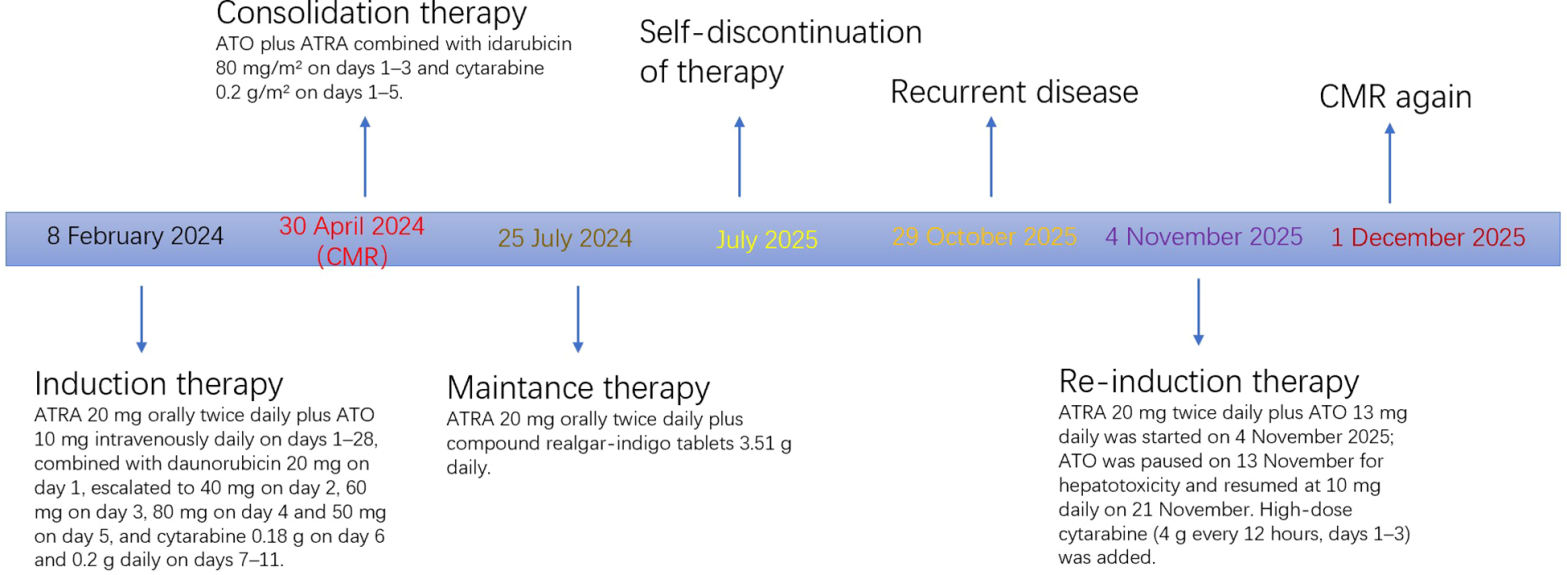
Treatment timeline of the patient.

However, the patient prematurely discontinued all maintenance therapy. Three months later, bone marrow evaluation revealed recurrent disease characterized by abnormal hypergranular promyelocytes with Auer rods, corroborated by flow cytometric detection of abnormal promyelocytes, and RT-PCR confirmation of *PML::RARA* fusion transcript positivity, consistent with hematologic relapse. He received re-induction with ATRA, ATO and DA regimens, and achieved CMR again (**Figure 2**).

## Patient perspective

The patient was a 34-year-old manual laborer with limited financial resources. Throughout the treatment, he continued working to support his medical expenses. Despite repeated counseling by the clinical team regarding the critical importance of maintenance therapy and the high risk of relapse upon treatment discontinuation, the patient self-discontinued all maintenance medications three months after their initiation. Following hematologic relapse, when allogeneic hematopoietic stem cell transplantation was recommended by the treating physicians as the optimal therapeutic strategy for high-risk relapsed APL, the patient declined this treatment option due to financial constraints and concerns regarding post-transplant care requirements, opting instead for the less expensive re-induction regimen.

## Discussion

APL is uniquely defined by the presence of *RARA* rearrangements, most commonly involving *PML*. While the vast majority of cases harbor canonical *PML::RARA* fusion transcripts detectable by standardized real-time quantitative PCR assays, rare or atypical breakpoints may escape routine molecular screening (7, 8). Such cases pose a significant diagnostic challenge, particularly when morphologic, immunophenotypic, and cytogenetic features strongly suggest APL.

Canonical *PML::RARA* fusion isoforms, including the long, short, and variant transcripts, account for the majority of APL cases and are reliably detected by PCR-based assays, with reported geographic and risk-related variability (9). However, these assays are inherently limited to predefined breakpoint regions and may fail to identify structurally atypical fusions, as illustrated by the present case.

Despite its atypical breakpoint structure, the novel fusion preserved critical functional domains required for leukemogenesis and retinoid responsiveness. Consistent with this molecular architecture, induction treatment with ATRA and ATO resulted in a rapid and deep molecular response, supporting the concept that therapeutic sensitivity in APL is determined by the presence of a functional *PML::RARA* fusion rather than by the exact exon junction involved (10).

Relapse occurred shortly after premature discontinuation of maintenance therapy. In addition to treatment interruption, the coexistence of *FLT3-ITD* and *WT1* mutations may have contributed to the aggressive clinical course and early recurrence, consistent with prior observations linking cooperating mutations to adverse outcomes in APL and AML (11, 12). FLT3-ITD is a frequently mutated gene in APL, reported in 30-40% of cases and associated with several poor-prognosis indicators such as high white blood cell counts, M3v variant morphology, and the bcr3 isoform (13, 14). WT1 mutations represent a prognostic risk factor in APL, and studies have demonstrated that high WT1 expression serves as a reliable biomarker for predicting subsequent molecular relapse in this disease (15, 16).

Standardized real-time quantitative PCR remains the gold standard for minimal residual disease monitoring in APL (6). However, this approach depends on prior knowledge of fusion breakpoints and may be inadequate for detecting rare or atypical *PML::RARA* variants. This case highlights the complementary value of RNA sequencing in diagnostic clarification and MRD assay design, enabling patient-specific molecular monitoring and potentially refining risk stratification in high-risk or conventional PCR-negative APL cases.

## Conclusion

We report the first case of APL harboring a previously unrecognized in-frame *PML* exon 8–*RARA* exon 3 fusion with partial deletion of *RARA* exon 3. Despite its atypical molecular architecture and high-risk clinical presentation, the leukemia remained responsive to ATRA and ATO, supporting the concept that therapeutic sensitivity in APL is determined by the presence of a functional *PML::RARA* fusion rather than by a specific exon junction. This case highlights an important diagnostic limitation of conventional PCR-based assays and underscores the value of RNA sequencing in resolving discordant molecular findings and guiding targeted therapy.

## Methods

Total RNA was extracted from bone marrow samples using the Lab-Aid 896 Genomic RNA Kit. The concentration and integrity of extracted nucleic acids were preliminarily assessed by Qubit 4.0 and Qseq400. Samples passing quality control were subjected to pre-library construction using the VAHTS Universal V8 RNA-seq Library Prep Kit for Illumina. Following pre-library generation, target region hybridization capture was performed by the Twist Universal Blocker, the Twist Standard Hyb and Wash Kit, combined with in-house designed hematologic malignancy-targeted probes from Xiehe Bojing Company. The captured target libraries were subsequently sequenced on the Illumina NovaSeq 6000 platform, with a guaranteed yield of ≥7 Gb per sample. Raw FASTQ data were processed by fastp (ref: fastp: an ultra-fast all-in-one FASTQ preprocessor) (version 0.23.2, https://github.com/OpenGene/fastp) to remove adapter sequences and low-quality reads. Candidate fusion gene pairs were identified by the open-source software Arriba (ref: Accurate and efficient detection of gene fusions from RNA sequencing data) (version v2.2.0, https://github.com/suhrig/arriba). Hotspot gene pairs were further verified against an internal fusion gene database, and all final confirmed fusion genes were manually inspected by the Integrative Genomics Viewer (IGV) (ref: Integrative Genomics Viewer (IGV): high-performance genomics data visualization and exploration) (version v2.16.2, https://www.igv.org/). Finally, patient-specific primers targeting the unique breakpoint (chr15:74327736::chr17:38504626) were designed for minimal residual disease monitoring, with a validated sensitivity of 10^-4^.

## Data Availability

The raw data supporting the conclusions of this article will be made available by the authors, without undue reservation.
